# A 6-week hip muscle strengthening and lumbopelvic-hip core stabilization program to improve pain, function, and quality of life in persons with patellofemoral osteoarthritis: a feasibility pilot study

**DOI:** 10.1186/s40814-018-0262-z

**Published:** 2018-04-06

**Authors:** Lisa T. Hoglund, Laura Pontiggia, John D. Kelly

**Affiliations:** 1 Department of Physical Therapy, Thomas Jefferson University, 901 Walnut Street, 5th Floor, Philadelphia, PA 19107 USA; 2 Department of Mathematics, Physics and Statistics, University of the Sciences, Philadelphia, PA USA; 3 Department of Sports Medicine and Orthopaedic Surgery, University of Pennsylvania, Philadelphia, PA USA

**Keywords:** Patellofemoral joint, Knee osteoarthritis, Patellofemoral osteoarthritis, Exercise, Treatment

## Abstract

**Background:**

Patellofemoral joint (PFJ) osteoarthritis (OA) is prevalent in middle-aged and older adults. Despite this, there are minimal studies which have examined conservative interventions for PFJ OA. Weakness of proximal lower extremity muscles is associated with PFJ OA. It is unknown if a hip muscle strengthening and lumbopelvic-hip core stabilization program will improve symptoms and function in persons with PFJ OA. This study examined the feasibility and impact of a 6-week hip muscle strengthening and core stabilization program on pain, symptoms, physical performance, peak muscle torques, and quality of life in persons with PFJ OA.

**Methods:**

Ten females with PFJ OA and ten age- and sex-matched controls participated in baseline tests. PFJ OA participants attended ten twice-a-week hip strengthening and core stabilization exercise sessions. Outcome measures included questionnaires, the Timed-Up-and-Go, and peak isometric torque of hip and quadriceps muscles. Data were tested for normality; parametric and non-parametric tests were used as appropriate.

**Results:**

At baseline, the PFJ OA group had significantly worse symptoms, slower Timed-Up-and-Go performance, and lower muscle torques than control participants. PFJ OA group adherence to supervised exercise sessions was adequate. All PFJ OA participants attended at least nine exercise sessions. Five PFJ OA participants returned 6-month follow-up questionnaires, which was considered fair retention. The PFJ OA participants’ self-reported pain, symptoms, function in daily living, function in sport, and quality of life all improved at 6 weeks (*P* < 0.05). Timed-Up-and-Go time score improved at 6 weeks (*P* = 0.005). Peak hip external rotator torque increased (*P* = 0.01). Improvements in pain and self-reported function were no longer significant 6 months following completion of the intervention.

**Conclusions:**

PFJ OA participants were adherent to the supervised sessions of the intervention. Improvement in symptoms, physical performance, and muscle torque were found after 6 weeks. Participant retention at 6 months was fair, and significant changes were no longer present. Our findings suggest that a hip strengthening and core stabilization program may be beneficial to improve symptoms, function, and physical performance in persons with PFJ OA. Future studies are needed, and additional measures should be taken to improve long-term adherence to exercise.

**Trial registration:**

ClinicalTrials.gov NCT02825238. Registered 6 July 2016 (retrospectively registered).

**Electronic supplementary material:**

The online version of this article (10.1186/s40814-018-0262-z) contains supplementary material, which is available to authorized users.

## Background

Knee osteoarthritis (OA) is a prevalent condition that causes significant pain, reduced quality of life, and disability [1–4]. More than 9 million adults in the USA have symptomatic knee OA, and this prevalence is increasing [3, 5]. Symptomatic knee OA age-standardized prevalence was reported to be 3.8% of the world’s population [2]. Knee OA and hip OA together are reported to be the 11th highest contributor to global disability [2].

The patellofemoral joint (PFJ) is a frequent site of knee OA [6–8]. The PFJ is the knee compartment most commonly affected by OA [8–10]. Patellofemoral joint OA was present in 39% of persons aged ≥ 30 years who reported knee pain [11]. The prevalence of PFJ OA may be even greater in persons with chronic PFJ pain. One study reported that PFJ OA was present in 69% of persons with chronic PFJ pain aged ≥ 40 years [8]. Isolated PFJ OA is associated with significant pain and disability, perhaps to an even greater degree than tibiofemoral joint (TFJ) OA [6, 12, 13]. The presence of even mild radiographic PFJ OA was associated with difficulty in activities requiring knee flexion during weight-bearing [12]. Activities reported as being most difficult for persons with PFJ OA include descending and ascending stairs, the sit-to-stand transfer, getting in and out of the bathtub, and getting in and out of a car [12]. Clinical features of PFJ OA may include anterior knee pain, a history of significant swelling, genu valgus malalignment, quadriceps muscle weakness, hip abductor weakness, abnormal pelvic and lower extremity (LE) biomechanics, and pain with PFJ compression [14–20]. Females with PFJ OA are reported to rate their pain as more severe compared to males with the same level of radiographic PFJ OA [21].

Management guidelines for knee OA have been published by several international organizations [22–26]. Two systematic reviews of OA management guidelines reported strong evidence for beneficial effects of the following non-pharmacologic interventions: exercise, education, self-management, weight loss, manual therapy, assistive walking devices, thermal modalities, and electrical stimulation modalities [22, 23]. Exercise and education were reported to be the interventions with the strongest evidence of efficacy and to be relatively inexpensive [23]. Aerobic, strengthening, aquatic, and general exercise have all been shown to be beneficial for persons with knee OA [24–28]. However, evidence for the most effective type of exercise for knee OA is currently lacking [29]. In addition, there are different knee OA phenotypes, for which optimal management may differ [30–34]. Patellofemoral OA may require different interventions than TFJ OA, given significant differences in joint biomechanics, cartilage structure, risk factors, muscle strength, and aggravating activities [15, 35, 36]. It has been proposed that a treatment program targeting the pathomechanics of PFJ OA may be more efficacious than a standard intervention for knee OA [37].

Despite the high prevalence, significant pain, and functional limitations caused by PFJ OA, there is a paucity of evidence for conservative treatment of this disabling condition. Studies examining exercise interventions for knee OA have frequently targeted the TFJ rather than the PFJ [38–41]. Currently, there are limited reports of non-surgical physical interventions specifically designed for PFJ OA [42–46]. Studies examining exercise as an intervention for PFJ OA have used a multimodal approach including manual therapy, education, and modalities [42, 45]. Quilty et al. [42] reported that a program of quadriceps strengthening exercise and medial patellar taping produced short-term improvement in knee pain and quadriceps strength compared to a control group that received no physiotherapy; however, these differences were no longer significant after 12 months. Crossley et al. [45] reported that a multimodal program of manual therapy, education, patellar taping, and exercise resulted in improved pain intensity and patient-perceived change compared to an education-only group. The exercise program used by Crossley et al. [45] included open- and closed-kinetic chain quadriceps strengthening, side lying hip abductor strengthening, and functional retraining of the vasti and/or hip abductor muscles during sitting, sit-to-stand, stepping up, and/or single-leg-squats. Differences in pain and patient-perceived change were no longer present 6 months following completion of the 3-month treatment session [45]. No differences between groups were found for self-reported function or actual physical performance in either study [42, 45]. Importantly, it is unknown which component(s) of these multimodal interventions were responsible for the short-term benefits.

Although limited evidence exists for the impact of exercise on pain, impairments, and disability in PFJ OA, substantial evidence is present for the beneficial effect of strengthening exercise for patellofemoral pain (PFP) in younger adults [47–56]. Patellofemoral pain in adolescence and young adulthood may be a possible predisposing factor to PFJ OA [57, 58]. Similar LE muscle weakness is present in both conditions, most notably the quadriceps, hip extensor, hip abductor, and hip external rotator muscles [14, 20, 59–63]. Altered pelvic and hip biomechanics during functional activities and impaired lumbopelvic-hip postural stability, i.e., core stability, has been reported in PFJ OA and PFP [19, 64–67]. Recent systematic reviews reported conflicting results regarding the superiority of quadriceps strengthening versus hip muscle strengthening in terms of pain relief in persons with PFP [53, 56, 68]. Alba-Martin et al. [53] reported that programs of hip abductor and hip external rotator muscle strengthening combined with quadriceps strengthening resulted in earlier pain relief compared to quadriceps strengthening alone in persons with PFP. A systematic review and meta-analysis of proximal muscle rehabilitation for PFP reported that programs including strengthening of the hip abductors, hip external rotators, and hip extensors were superior to quadriceps strengthening programs for pain reduction in the short and medium terms as well as improved function in the medium term [68]. Thomson et al. [56] reported that hip-focused and knee-focused strengthening programs were both effective to reduce PFP. But a program of combined hip and quadriceps muscle strengthening was superior to quadriceps strengthening alone in terms of reduced pain intensity and improved function in the short, medium, and longer terms for persons with PFP [68]. Hip strengthening programs for PFP frequently include activities to promote core stabilization, including many studies in the systematic review by Thomson et al. [51, 52, 56, 69–74] More recently, females with PFP treated with core stabilization exercises combined with knee-focused exercise resulted in less pain, better self-reported function, and better physical performance on the Timed-Up-and-Go and a hop test compared to a group treated with only knee-focused exercise [75].

Proximal LE strength and core stability have been proposed to be important as a stable foundation for LE movement in closed chain functional activities [76]. A recent expert consensus statement reported that persons with PFP have reduced hip and trunk muscle strength as well as altered trunk and LE kinematics during functional activities [77]. It is proposed that altered trunk and hip kinematics may cause altered tibiofemoral and/or patellar kinematics, with resultant increased PFJ stress and PFP [77]. Strengthening hip muscles and improving lumbopelvic-hip core stability may reduce reliance on the quadriceps muscle during flexed knee activities, thus reducing PFJ stress and PFP [36]. Reduction of PFJ stress may be critical to achieve pain relief and facilitate exercise participation in persons with PFJ OA [68]. It is currently unknown if a program of strengthening exercises for the hip external rotators, hip abductors, and hip extensors combined with lumbopelvic-hip core stabilization exercises and education to promote core stability will be effective at decreasing knee pain, improving function, and improving quality of life (QOL) in persons with painful PFJ OA.

The aim of this study was to determine the feasibility and effect of a program including hip and core muscle strengthening, core stabilization, and LE alignment neuromuscular education for persons with PFJ OA on knee pain, self-reported function, physical performance, and QOL compared to baseline.

## Methods

A pre-post intervention design was used for this proof of concept, feasibility pilot study [78]. The primary objectives were to (1) estimate adherence to the supervised exercise program, (2) estimate long-term retention rate and willingness to respond to a 6-month follow-up, and (3) establish the viability of the treatment model through the impact on participants’ pain ratings, activity of daily living (ADL), and global rating of change after the 6-week supervised program. Secondary objectives of this study were to (1) assess the impact of the intervention on physical performance, (2) determine the long-term benefits of the intervention in terms of self-reported pain and function at a 6-month follow-up, and (3) assess the impact of the intervention on hip and knee muscle torques.

### Participants

Ten persons with painful PFJ OA were recruited from an orthopedic surgeon (JK) at the Department of Sports Medicine at the University of Pennsylvania and in response to recruitment posters placed at the University of the Sciences and the local community. Potential participants were screened for eligibility with a questionnaire by the lead investigator (LH). Persons who appeared to satisfy all study inclusion and exclusion criteria for the PFJ OA group were screened for the presence of radiographic PFJ OA on knee diagnostic imaging studies by an orthopedic surgeon (JK) trained in use of the Osteoarthritis Research Society International (OARSI) grading scale [79, 80]. Potential participants who passed both phases of screening were invited to participate in the study (PFJ OA group). An additional cohort of 10 age- and sex-matched asymptomatic control participants were recruited for comparison to baseline PFJ OA group status (control group). Testing and the intervention took place in the research laboratories at the University of the Sciences Department of Physical Therapy.

Inclusion criteria for the PFJ OA group were (1) aged 35–70 years, (2) diagnosed by a medical physician with knee OA in one or both knees with symptoms primarily in the anterior knee, (3) an OARSI OA grade of 1 or more at the PFJ on diagnostic images (0 = none, 1 = mild/possible, 2 = moderate/definite, 3 = severe) [79, 80], (4) presence of ≥ 2 of the following: pain produced by descending steps, ascending steps, sit-to-stand, squatting, kneeling, prolonged knee flexion, increased activity (e.g., hiking); morning stiffness < 30 min; stiffness after sitting ≥ 30 min; or a history of patellar subluxation or dislocation in the past [20, 58, 81]. Exclusion criteria were (1) presence of other conditions that may cause knee pain, (2) neurologic or musculoskeletal conditions that may alter LE strength or movement, (3) history of a knee joint fracture, (4) pregnancy, (5) knee injections within the previous 3 months, and (6) inability to understand English. Control group inclusion criteria were (1) aged 35–70 years, (2) no known diagnosis of knee OA, (3) pain-free LE at time of enrollment and for 1 week prior. Exclusion criteria for the control group included (1) all exclusion criteria for the PFJ OA group, (2) presence of ≥ 2 of the following: a history of patellar subluxation or dislocation, and pain produced by descending steps, ascending steps, sit-to-stand, squatting, kneeling, and prolonged knee flexion. Control group participants were matched to PFJ OA group participants according to sex and age (± 10 years).

All participants signed a written informed consent prior to inclusion in the study, and the research was performed in accordance with the Declaration of Helsinki. The study was approved by the University of the Sciences Institutional Review Board (IRB) and by the University of Pennsylvania IRB, Philadelphia, Pennsylvania, USA.

### Procedure

Potential PFJ OA group participants were identified for possible eligibility by an orthopedic surgeon from clinical practice (JK). Additional potential participants responded to recruitment posters. All participants of both groups were screened with a screening questionnaire. Diagnostic imaging studies of the knees of potential PFJ OA group participants were reviewed and graded to confirm the presence of at least OARSI Grade 1 PFJ OA. Following admission to the study, subjects read the informed consent form, had any questions regarding the study procedures answered, and gave written informed consent. Participants rated their knee pain intensity with an 11-point numeric pain rating scale (NPRS), with 0 = no pain and 10 = worst pain imaginable. The NPRS is reported to be reliable and valid in persons with knee OA [82, 83]. A change of 2 points on the NPRS is clinically significant in persons with chronic pain [84]. Participants completed the Knee Injury and Osteoarthritis Outcome Score (KOOS), a patient-reported measure of symptoms and function in persons with knee injury and knee OA [85]. The KOOS is reliable, valid, and responsive to change for persons with knee OA [86]. The KOOS contains five subscales: pain, symptoms, ADL, sport/recreation, and QOL. Each subscale includes several items, scored with a 5-point Likert-scale score (0 = no pain/full function, 4 = extreme pain/worst function). The mean subscale score is divided by 4; the result is multiplied by 100; this figure is subtracted from 100 to determine the resultant subscale score (100 = least pain/best function possible). The KOOS subscale minimum clinically important differences (MCID) were reported to be 8–10 points for each subscale in persons with knee injury or osteoarthritis [87]. More recently, KOOS subscale changes of 14.3–19.6 points were reported to indicate true change in younger patients with knee injury treated conservatively [86]. For the purposes of this feasibility study, the KOOS pain and ADL subscale scores were considered most critical.

Participants were examined to determine height, weight, knee joint line height, and tenderness to palpation of the patella and patellar tendon. Participants’ physical performance was assessed with the Timed-Up-and-Go (TUG), a timed test of a person’s ability to stand from a chair, walk 3 m, turn, walk back, and sit back down [88, 89]. The TUG includes the sit-to-stand transfer, one of the activities reported to be most impaired in persons with PFJ OA, perhaps due to high PFJ stress from strong quadriceps contraction with the knee in a highly flexed position [12, 13]. Persons with bilateral PFJ OA required significantly longer time to complete the TUG compared to a pain-free control group without radiographic PFJ OA [13]. The TUG is valid and responsive to change in persons with knee OA and is recommended by OARSI as one of the core physical performance outcome measures in persons with knee OA [89–91]. The MCID for the TUG in persons with knee OA is reported to be 0.8–1.4 s [90]. Participants performed one practice trial followed by three timed trials of the TUG. The average of three trials was used as the result. Participants reported a pre- and post-TUG NPRS for knee pain [92].

Strength of the knee extensor, hip abductor, hip extensor, and hip external rotator muscles were examined with a peak isometric muscle torque test using the BTE Primus RS™ dynamometer (BTE Technologies, Inc., Hanover, MD, USA) [93–96]. The Primus RS™ was shown to have excellent intrarater test-retest reliability for LE muscle tests using this methodology [96]. Peak torques were normalized according to height and weight with the formula Nm torque/(kg mass × height in meters) [97]. Muscle torque tests were three maximal effort isometric trials, 3 s in length, with a rest of 30 s between trials. The average of three trials was used as the result. Stabilizing straps were used to maintain participants in the test position. Participants performed two submaximal practice trials prior to recorded trials to familiarize them with the test. Participants were instructed to exert maximal effort and were verbally coached to push as hard as they could for each recorded trial. Trials were repeated if compensatory motion was observed or if the coefficient of variation for the three recorded trials was > 10% [96]. The order of muscle torque testing was randomized with a random number generator to prevent an order effect.

**Fig. 1:**
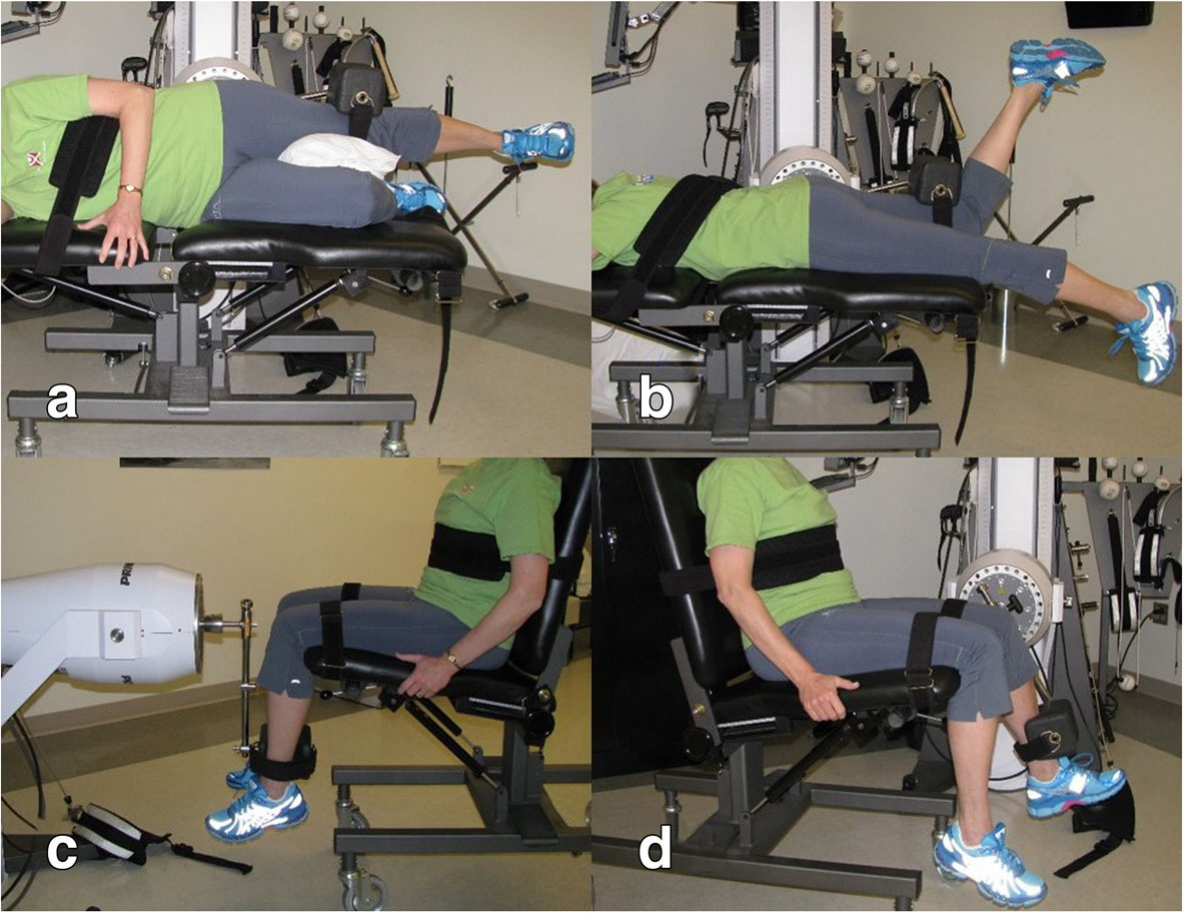
Peak isometric muscle torque testing positions. **a–d** The positions of participants during testing. **a** Hip abductor test position. **b** Hip extensor test position. **c** Hip external rotator test position. **d** Knee extensor test position

Hip abductor test position had the participant side lying with the tested LE uppermost with the hip and knee in the anatomical neutral position, opposite LE hip and knee flexed to approximately 45° each [93–95]. The trunk was stabilized with straps, dynamometer axis of rotation was opposite the uppermost gluteus maximus at the level of the greater trochanter, and the resistance pad was positioned on the distal lateral thigh 2.5 cm proximal to the lateral femoral epicondyle (Fig. 1a). Hip extensor test position had the participant prone with the tested LE at anatomical neutral hip position, tested knee flexed to approximately 80°, dynamometer axis of rotation opposite the tested LE greater trochanter, stabilization straps at the trunk and contralateral thigh, and the resistance pad at the posterior distal thigh of the tested LE (Fig. 1b) [93–95]. Hip external rotator test position had the participant seated with the hip at 90° flexion and neutral frontal and transverse plane rotation, knee flexed to 90°, dynamometer axis of rotation opposite the anterior knee in line with the femoral shaft longitudinal axis, stabilization straps at the distal thigh and trunk, and the resistance pad positioned on the medial lower leg 5 cm proximal to the medial malleolus (Fig. 1c) [96]. Knee extensor peak torque test position was with the participant seated with the hip at 90° flexion and neutral frontal and transverse plane position, knee flexed to 60°, dynamometer axis of rotation opposite the lateral femoral epicondyle, stabilization straps at the distal thigh and trunk, and the resistance pad positioned on the anterior lower leg 5 cm proximal to the lateral malleolus (Fig. 1d) [98].

### Rehabilitation protocol

Patellofemoral OA group subjects participated in a supervised exercise program. This novel intervention included progressive strengthening of the hip and core muscles, biofeedback to promote lumbopelvic-hip core stability, and neuromuscular reeducation to promote proper LE alignment during functional activities. Since the hip abductor, hip extensor, and hip external rotator muscles have been shown to be weak in persons with PFJ OA and in younger persons with PFP, the program initially focused on exercises to target strengthening those muscle groups. Treatment began primarily in a recumbent position to minimize recruitment of the quadriceps muscle and to minimize PFJ reaction forces and PFJ stress [70]. During the last 2 weeks of the program, exercises progressed to a more functional, weight-bearing position, including training in proper lumbopelvic-hip and knee alignment to avoid dynamic genu valgus and contralateral pelvic dropping. Quadriceps muscle strengthening likely occurred during weight-bearing exercises, but no exercises specifically targeting the quadriceps were utilized.

The rehabilitation program consisted of an initial evaluation session, 10 supervised exercise sessions over the course of 5–6 weeks, and a re-evaluation session. Sessions were provided two times per week for 6 weeks, with one exercise session in weeks 1 and 6 and two exercise sessions in weeks 2–5. Participants were allowed up to 8 weeks if necessary to complete all sessions, depending upon availability and scheduling. Exercise sessions were approximately 60-min duration. The supervised exercise program was based on progressive strengthening of hip and core muscles, lumbopelvic-hip core stabilization and endurance during destabilizing activities, stretching of LE muscles reported to be tight in PFP [99], auditory and visual feedback to improve core stabilization during functional activities, and neuromuscular reeducation to avoid excessive knee abduction, hip adduction, and hip internal rotation during weight-bearing activities and ADL. Participants were instructed to maintain a level pelvis during single leg stance activities, to keep their knee centered over their second toe, and to avoid allowing their knees to move excessively inwards or outwards during weight-bearing exercises and activities (sit-to-stand practice, lunges, etc.). Exercise sessions were supervised by licensed physical therapists and by student physical therapists trained in the protocol by the principal investigator. Resistance load was determined by participants’ ability to perform exercises with good core stabilization and LE control, without increase in knee pain, and without production of pain in other body regions [45, 100]; this was determined by the supervising physical therapist or principal investigator. Participants were instructed in a home exercise program to supplement the supervised sessions and given written instructions, an exercise diary, and therapeutic resistance tubing of various levels, as appropriate. The exercise program changed on a weekly basis throughout the supervised exercise portion of the program and participants were given new written, pictorial, and verbal instructions each week. Participants were instructed in a maintenance home exercise program at the conclusion of the supervised sessions. An additional file shows the exercise program in more detail (see Additional file 1).

Outcome measures at the 6-week follow-up of the PFJ OA group included (1) the KOOS questionnaire; (2) the TUG physical performance test; (3) tests of peak isometric torque of the hip abductors, hip extensors, hip external rotators, and knee extensors; and (4) a global rating of change (GRC). The GRC is a general self-report measure of overall change scored from − 5 to 5 with anchors of “very much worse” (− 5), “unchanged” (0), and “completely recovered” (5) [101]. A change of 2 points on this 11-point scale is considered to be the MCID [101]. Participants were asked to rate their change with respect to their PFJ OA from the time immediately before beginning the supervised exercise program to the conclusion of the 6-week supervised exercise program.

Patellofemoral joint OA group participants continued the maintenance home exercise program for 6 months following completion of the supervised exercise program. Exercise reminders were sent to participants as mobile device text messages or email messages on a monthly-bimonthly basis. Six months following completion of the supervised exercise program, PFJ OA group participants were mailed a KOOS questionnaire and asked to complete this and return it with the exercise diary.

### Statistical analysis

Descriptive statistics were used to summarize the data. Quantitative data were reported as percentages, numeric counts, mean ± standard deviation (SD), or median and interquartile range (IQR). Exploratory comparisons between the PFJ OA group and the control group at baseline were conducted using either a *t* test, if the normality assumption was met by the data, or Wilcoxon Rank Sum test, otherwise. An exploratory analysis was also performed to assess the changes in outcome variables from baseline to 6 weeks for the PFJ OA group using paired *t* tests, if the normality assumption was met by the data, or Wilcoxon signed rank tests, otherwise. The baseline to 6-month and 6-week to 6-month changes in outcome variables for the PFJ OA group were assessed with Wilcoxon signed rank tests. A *P* value less than 0.05 was considered statistically significant. All statistical analyses were performed using SAS version 9.4 (SAS Institute, Inc. Cary, NC, USA).

## Results

All 20 study participants were female. Participants ranged in age from 37 to 65 years (PFJ OA group 41–64 years, control group 37–65 years). There was no significant difference in age between groups (Table 1). The PFJ OA group was significantly heavier and had a significantly greater body mass index (BMI) than the control group (Table 1). Radiographic OA severity in the PFJ OA group ranged from mild to moderate for the PFJ (Table 2). Mild to moderate TFJ OA was present in some of the PFJ OA participants as well (Table 2). At baseline, the PFJ OA group had significantly greater pain compared to the control group, as measured by the NPRS and the KOOS pain subscale (Table 3). The PFJ OA group had greater symptoms, lower function in ADL and sport, and lower QOL at baseline versus the control group, as measured with the KOOS (Table 3). The PFJ OA group also had significantly lower peak isometric muscle torque at baseline compared to the control group (Table 3). PFJ OA group participants required longer time to complete the TUG at baseline than did control group participants, and reported significantly greater pre- and post-TUG pain (Table 3).

**Table 1:** Demographic characteristics of participants (*n* = 20, 100% female)

	PFJ OA group	Control group	*P* value
Age (years)	50 (46, 56)	52 (49, 56)	0.6415
Height (m)	1.64 (1.60, 1.69)	1.67 (1.58, 1.73)	0.8677
Weight (kg)	102.35 (75.70, 112.30)	66.55 (60.80, 70.70)	*0.0007*
BMI	33.04 (28.28, 41.02)	23.41 (22.51, 27.09)	*0.0021*
History of patellar subluxation or dislocation (*n*)	1 (dislocation)	0	–

Data are expressed as median (IQR). Values in italics indicate significant differences at the 0.05 significance level in the exploratory analyses (Wilcoxon Rank Sum test)

*n* number, *PFJ OA* patellofemoral joint osteoarthritis, *BMI* body mass index, *IQR* interquartile range

**Table 2:** Diagnostic imaging characteristics for patellofemoral joint osteoarthritis group participants, frequency (*n* = 10, 100% female)

Patellofemoral joint					
OARSI grade		0	1	2	3
JSN	Medial	5	2	2	0
	Lateral	5	4	0	0
Marginal osteophytes	Medial	3	3	0	0
	Lateral	2	3	1	0
	Superior	3	6	1	0
	Inferior	6	4	0	0
Subchondral bony sclerosis	Medial	3	2	0	0
	Lateral	2	2	1	0
Subluxation	Medial	6	0	0	0
	Lateral	4	2	0	0
Tibiofemoral joint					
OARSI grade		0	1	2	3
JSN	Medial	6	2	1	0
	Lateral	6	3	0	0
Marginal osteophytes	Medial	8	0	1	0
	Lateral	8	1	0	0
Subchondral bony sclerosis	Medial	4	5	0	0
	Lateral	8	1	0	0
		None	Varus	Valgus	
Tibiofemoral malalignment		6	2	1	–

Total may not equal total number participants due to missing data/radiographic views for some participants

*n* number, *OARSI* Osteoarthritis Research Society International (0 = none, 1 = mild, 2 = moderate, 3 = severe), *JSN* joint space narrowing

**Table 3:** Baseline test results, comparison between groups

	PFJ OA group	Control group	*P* value
KOOS Pain (0–100)	63.89 (47.22, 66.67)	100 (100, 100)	*< 0.0001*
KOOS Symptom (0–100)	60.71 (53.57, 67.86)	98.21 (92.86, 100)	*< 0.0001*
KOOS ADL (0–100)	68.38 (47.06, 76.47)	100 (100, 100)	*< 0.0001*
KOOS Sport/Rec (0–100)	30 (20, 50)	100 (100, 100)	*0.0001*
KOOS QOL (0–100)	37.50 (25, 43.75)	100 (100, 100)	*< 0.0001*
NPRS (0–10)	3 (3, 6)	0 (0, 0)	*0.0001*
Hip Ext Rot Torque	0.15 ± 0.02	0.24 ± 0.07	*0.0010*
Hip Abd Torque	0.33 (0.31, 0.41)	0.48 (0.43, 0.59)	*0.0007*
Hip Ext Torque	0.11 (0.07, 0.21)	0.27 (0.20, 0.43)	*0.0089*
Knee Ext Torque	0.43 (0.37, 0.62)	0.77 (0.72, 0.87)	*0.0089*
TUG (s)	7.42 (6.46, 8.55)	5.88 (5.09, 5.96)	*0.0336*
Pre-TUG Pain (0–10)	2.00 (0.00, 4.00)	0 (0, 0)	*0.0031*
Post-TUG Pain (0–10)	2.00 (1.00, 4.00)	0 (0, 0)	*0.0007*

Data are expressed as median (IQR) except for Hip Ext Rot Torque, which is expressed as mean ± SD. Values in italics indicate significant differences at the 0.05 significance level in the exploratory analyses (Wilcoxon Rank Sum test [median {IQR} results] or two-sample *t* test [mean ± SD] results). KOOS scored from 0 to 100, 100 = best status. NPRS scored from 0 to 10, 10 = worst pain

*PFJ OA* patellofemoral joint osteoarthritis, *KOOS* Knee Injury and Osteoarthritis Outcome Score, *ADL* activity of daily living, *Rec* recreation, *QOL* quality of life, *NPRS* numeric pain rating scale, *Ext Rot* external rotator, *Abd* abductor, *Ext* extensor, *TUG* Timed-Up-and-Go, *IQR* interquartile range, *SD* standard deviation

Eighteen persons were screened for participation in the PFJ OA group exercise intervention; 10 of these 18 potential subjects participated in the study (55.6% participant yield). Screen failures or reasons for non-participation for the PFJ OA group included viscosupplementation knee injection within the previous 3 months (1 potential participant), neuromuscular comorbidity (1), history of bony patellofemoral realignment surgery (1), radiographs older than 1 year (1), no physician diagnosis of knee OA (1), and declined/did not contact the principal investigator (3). The intervention was acceptable to persons with PFJ OA: only 3 of 13 potential PFJ OA group participants who met all inclusion and exclusion criteria declined to participate; the PFJ OA participation rate for those meeting all study criteria was 76.9%. Adherence to the supervised exercise program was good: 9 participants attended all 12 supervised sessions and 1 participant attended 11 sessions, only missing 1 exercise session. There were no adverse reactions attributable to the exercise intervention.

The long-term retention rate over 6 months following the intervention and willingness to respond to a 6-month follow-up was fair-poor. Only 5 participants returned the KOOS questionnaire for a 50.0% retention rate, which was considered fair. One participant dropped out of the study after completion of the 6-week supervised exercise program but before the 6-month follow-up since she elected to have a surgical procedure on the index knee (reported when contacted with an exercise reminder). Excluding the participant who had surgery, the 6-month follow-up retention rate for completion of the KOOS was 55.6%. Only 2 participants returned the exercise calendar, which was considered poor. One exercise calendar was not completed at all while 1 exercise calendar was completed for the 6-week supervised exercise sessions and for the 6 months of follow-up (3–5 sessions per week during the 6-week supervised session time period and 3 sessions per week during the 6-month follow-up time period). It is unknown if participants who did not return their exercise calendar diaries completed them.

The PFJ OA group significantly improved in the short term on the NPRS and on all KOOS subscales; all of the baseline to 6-week changes in these variables surpassed the MCID values (Table 4). The PFJ OA group GRC immediately following the intervention was 2.70 ± 0.67 (mean ± SD), which is higher than the MCID of 2 points [101]. Physical performance on the TUG significantly improved at the 6-week follow-up compared to baseline; the improvement in time surpassed the MCID of 0.8 s (Table 5). Peak isometric muscle torque significantly improved for the hip external rotators (Table 5). Isometric muscle torque increased for the other muscle groups, but differences did not reach statistical significance (Table 5). Baseline to 6-week median changes in hip external rotator and hip extensor peak isometric muscle torque were both greater than 14% increase, the reported standard error of measurement for hip isometric muscle strength and recommended value for minimum important difference for the hip muscles [102]. The median baseline to 6-week change in knee extensor peak isometric muscle torque was a 14% increase, which is below the recommended minimum knee extensor strength gain necessary to result in reduced disability (40% gain) and to result in reduced pain (30% gain) [103].

**Table 4:** PFJ OA participant pain rating and KOOS scores: baseline, 6-week follow-up, and change (*n* = 10)

	Baseline	6-week follow-up	Participant score change from baseline	*P* value
NPRS (0–10)	3 (3, 6)	1 (0, 3)	− 2.50 (− 3, − 2)	*0.0195*
KOOS Pain (0–100)	63.89 (47.22, 66.67)	77.78 (69.44, 86.11)	16.67 (5.56, 25.00)	*0.0039*
KOOS Symptom (0–100)	60.71 (53.57, 67.86)	75.00 (57.14, 89.29)	14.29 (10.71, 17.86)	*0.0156*
KOOS ADL (0–100)	68.38 (47.06, 76.47)	83.82 (79.41, 94.12)	15.44 (14.71, 17.65)	*0.0020*
KOOS Sport/Rec (0–100)	30 (20, 50)	57.50 (50.00, 75.00)	25.00 (0, 45.00)	*0.0195*
KOOS QOL (0–100)	37.50 (25, 43.75)	50.00 (31.25, 62.50)	15.63 (6.25, 25.00)	*0.0195*

Data are expressed as median (IQR). Values in italics indicate significant change at the 0.05 significance level in the exploratory analyses (Wilcoxon Signed Rank test). KOOS scored from 0 to 100, 100 = best status. NPRS scored from 0 to 10, 10 = worst pain

*PFJ OA* patellofemoral joint osteoarthritis, *n* number, *NPRS* numeric pain rating scale, *KOOS* Knee Injury and Osteoarthritis Outcome Score, *ADL* activity of daily living, *Rec* recreation, *QOL* quality of life, *IQR* interquartile range

**Table 5:** PFJ OA participant peak isometric muscle torque and TUG at baseline and 6-week follow-up

	Baseline	6-week follow-up	Change from baseline	*P* value
Hip Ext Rot Torque	0.16 (0.13, 0.16)	0.19 (0.17, 0.24)	0.04 (0.02, 0.08)	*0.0137*
Hip Abd Torque	0.33 (0.31, 0.41)	0.34 (0.28, 0.46)	− 0.04 (− 0.11, 0.07)	0.6953
Hip Ext Torque	0.11 (0.07, 0.21)	0.16 (0.12, 0.38)	0.06 (−0.01, 0.10)	0.1309
Knee Ext Torque	0.43 (0.37, 0.62)	0.68 (0.37, 0.75)	0.06 (−0.03, 0.24)	0.3223
TUG (s)	7.61 ± 1.70	6.63 ± 1.36	− 0.98 ± 0.84	*0.0052*
Pre-TUG Pain (0–10)	2.00 (0.00, 4.00)	0 (0, 3)	− 1 (−3, 0)	0.1719
Post-TUG Pain (0–10)	2.00 (1.00, 4.00)	0 (0, 3)	− 2 (−3, 0)	0.1250

Data are expressed as median (IQR) except for TUG which is expressed as mean ± SD. Values in italics indicate significant change at the 0.05 significance level in the exploratory analyses (Wilcoxon Signed Rank test [median {IQR} results] or paired *t* test [mean ± SD] results)

*PFJ OA* patellofemoral joint osteoarthritis, *Ext Rot* external rotator, *Abd* abductor, *Ext* extensor, *TUG* Timed-Up-and-Go, *IQR* interquartile range, *SD* standard deviation

Long-term response to the exercise intervention was measured with the KOOS questionnaire. Although the 5 participants who completed and returned the KOOS still had subscale scores greater than baseline, the scores had declined since the 6-week follow-up. Change in KOOS subscale scores between the 6-month follow-up and baseline was no longer significant for any subscale (median [IQR] KOOS subscale change baseline to 6-month follow-up: pain = 13.89 [0, 25.00], symptoms = 10.71 [10.71, 21.43], ADL = 11.76 [5.88, 17.65], sport/recreation = 15.00 [0, 40.00], QOL = 25.00 [0, 37.50]; all *P* > 0.05). All KOOS subscale change scores from baseline to 6-month follow-up were still greater than the MCID of 8–10 points, however. Change in KOOS subscale scores between the 6-week follow-up and the 6-month follow-up was no longer significant for any subscale (median [IQR] KOOS subscale change 6-week follow-up to 6-month follow-up: pain = 0.00 [− 5.56, 2.78], symptoms = − 3.57 [− 10.71, 0.00], ADL = − 4.41 [− 9.74, − 1.47], sport/recreation = 0 [− 5.00, 5.00], QOL = − 6.25 [− 18.75, − 6.25]; all *P* > 0.05).

## Discussion

This study is the first to our knowledge to examine the effect and feasibility of a hip and core muscle strengthening and stability exercise program as an intervention for persons with painful PFJ OA. Participants found the supervised exercise program acceptable, with all PFJ OA group participants attending the baseline assessment, 6-week follow-up session, and at least nine of ten exercise sessions. The retention rate for the 6-month follow-up was not as favorable with only 50% of the PFJ OA group returning a completed KOOS questionnaire and only 20% returning an exercise calendar diary. Only 1 of these exercise diaries was completed. It is unknown if participants who did not return their exercise diaries completed them. Participants who returned the KOOS at the 6-month follow-up generally had lower pain and symptoms and better function than prior to the exercise intervention, but their self-reported status was no longer significantly improved compared to baseline. However, the baseline to 6-month follow-up changes were still greater than the MCID for all subscales of the KOOS, indicating that the changes may have been clinically important [87]. It is possible that the positive change from baseline to the 6-months follow-up would have been statistically significant if all participants returned the KOOS. The fair-poor retention over the 6 months of unsupervised exercise may be an indication of low adherence to the exercise program, which has been reported to be common for persons with knee OA [104].

The PFJ OA group participants appeared to respond to the supervised hip and core muscle strengthening and core stabilization intervention, with significant short-term improvements reported for pain, symptoms, ADL, sport/recreation, and QOL at the conclusion of the 6-week supervised program. Changes on the KOOS subscales all surpassed the MCID of 8–10 points and were all in the range reported to indicate true change in younger patients treated conservatively (14.3–19.6 points) [86, 87]. In addition, participants rated their overall status at the 6-week follow-up as partially recovered, with the mean GRC score exceeding the MCID. Importantly, the exercise intervention resulted in improvements in the PFJ OA group’s physical performance and muscle strength as well. PFJ OA group participants completed the TUG in less time at the 6-week follow-up compared to baseline. It is recommended to collect pain ratings with the TUG time score for a more complete measure of mobility [92]. The pre- and post-TUG NPRS decreased; however, the change was not statistically significant. This lack of significance may be due to the relatively low baseline TUG NPRS and the small sample size. Participants increased their hip muscle strength, as evidenced by significantly increased isometric hip external rotator muscle torque. Hip extensor, hip abductor, and knee extensor isometric muscle torque also increased, though not reaching statistical significance. Improvement in hip external rotator and hip extensor isometric muscle torques increased by a median of 25 and 55%, respectively; both of these percentage improvements were well above the recommended minimum important difference of 14% for hip muscles [102]. Improved TUG performance may have been due to increased LE strength, decreased pain, neural adaptation, or a combination of these factors [105].

Participants in the PFJ OA group had significantly worse pain, symptoms, QOL, and function compared to the asymptomatic control group, consistent with earlier studies [12, 13]. Our findings that persons with PFJ OA have significantly worse physical performance as measured by the TUG compared to age- and sex-matched controls is also consistent with an earlier study [13]. Time score for the TUG for PFJ OA participants was similar (median 7.42 s for the current study, 7.5 s for the earlier study), but the current control group median TUG time was lower than an earlier study (median 5.88 s current study, 6.4 s earlier study) [13]. The difference may be due to the lower median age in the current study versus the earlier study (median 52 years and 57 years, current and earlier study, respectively) [13]. Our study adds to limited reports of reduced physical performance in persons with PFJ OA and demonstrates the usefulness of the TUG as a physical performance measure in this population [91]. The TUG may be particularly useful as a physical performance measure in persons with PFJ OA since it includes sit to stand, an activity requiring strong quadriceps contraction with the knee in a highly flexed position and is estimated to result in PFJ reaction forces of 6.70 times body weight [106].

A surprising finding was that the hip extensors and hip abductors did not significantly increase in peak isometric torque, despite a focus of the program on strengthening these muscle groups. This may be due to the small sample size and relatively high variability of participant torque change. Participants may also have learned improved core stabilization, thus improving isolated muscle recruitment. One component of this novel intervention was neuromuscular education in proper lumbopelvic-hip core stabilization during exercise. Some hip abductor and hip extensor strengthening exercises were similar to the isometric torque test positions for these two muscle groups. During supervised exercise sessions, participants were given verbal, written, and tactile feedback to isolate gluteus medius and gluteus maximus recruitment, and avoidance of pelvic or trunk motion. It may be that participants focused on isolated recruitment of gluteus medius and gluteus maximus muscles during 6-week hip abductor and hip extensor tests, respectively. In contrast, at baseline, they may have recruited secondary muscles to assist with performance of these tests. This may explain the apparent lack of significant short-term hip abductor muscle torque change, despite participants’ ability to perform exercise with heavier resistance loads. Since we did not collect electromyographic data during testing, we are unable to comment on a change in muscle recruitment following the supervised exercise intervention.

The lack of significant baseline to 6-week follow-up change in peak isometric muscle torque for the hip extensors, hip abductors, and knee extensors may have been due to insufficient overload of these muscles. We did not use specific repetition maximum (RM) testing to calculate the exercise load intensity; rather, we focused on movement control prior to adding resistance. The American College of Sports Medicine (ACSM) recommends use of testing to determine a healthy person’s 1 RM (1-RM) level and then performing 2–4 sets of 8–12 repetitions of 60–80% of the 1-RM to improve muscle strength [107]. The ACSM recommends beginning exercise intensity at 60–70% 1-RM or use of a Rating of Perceived Exertion Scale score of 5–6 (scale of 0–10, 10 = highest exertion) in persons who are older, very deconditioned, or susceptible to musculotendinous injuries [107]. The lower exercise intensity may be most appropriate for persons with painful PFJ OA who are often deconditioned, as comparison of the PFJ OA group muscle torque baseline scores to the control group baseline scores demonstrated (Table 3). An additional baseline exercise intensity level which could be used is the 10 RM (10-RM) value; this is recommended in a systematic review reporting principles of resistance training for persons with knee OA [105]. Although we progressed exercise intensity through use of cuff weights, greater resistance in therapeutic resistance bands, and altering body position, we may not have overloaded the muscles sufficiently to cause increased strength. We did follow ACSM resistance training criteria for recommended number of sets, number of repetitions, exercise session duration, frequency of moderate exercise sessions, and progression [107]. The lack of following ACSM criteria for resistance exercise intensity in our study is consistent with the findings of a systematic review of resistance training exercise studies for persons with knee OA [105].

The findings of significant short-term improvement in pain as measured with the NPRS and KOOS are consistent with earlier studies that included LE strengthening as part of a multimodal conservative intervention for PFJ OA [42, 45]. The current study, which was solely an exercise intervention, differed from earlier studies in that it resulted in significant improvements in self-reported ADL, QOL, and physical performance at the end of 6 weeks. Earlier studies examining conservative, multimodal approaches for persons with PFJ OA did not show any change in ADL or QOL [42, 45]. Importantly, our study is the first to report short-term improvement in participants’ objective physical performance, time to complete the TUG. It may be that the most important factor in a PFJ OA rehabilitation program is a proximal LE strengthening and core stabilization program. Earlier multimodal approaches had less face-to-face time spent by the physical therapist with participants and part of the time was allocated to patellar taping and/or patellar mobilization. Supervised sessions in the study by Quilty et al. [42] included only 4.5 h over 10 weeks and the study by Crossley et al. [45] included only 8 h over 12 weeks. The current study included 10 h of supervised sessions over 5–6 weeks, which were entirely exercise and neuromuscular reeducation of LE movement and lumbopelvic-hip core stability. Short-term improvements in ADL, physical performance, and QOL in our study may have been due to the greater volume of supervised exercise received by participants [108]. One recommended method to increase strength and improve muscle adaptations to exercise is to increase the volume of exercise per session by increasing the number of exercises or the number of sets of repetitions performed [108]. It may be that the most important factor for optimal patient outcomes is the amount of time spent in strengthening exercise and neuromuscular reeducation for proper LE alignment and core stabilization during functional activities. The combination of a greater volume of exercise during exercise sessions combined with proper exercise intensity to overload the muscle will result in significant muscle strengthening and even greater improvement in function and reduction in pain [105, 107].

This study has several limitations. There was a small sample size, as appropriate for a feasibility study; therefore, firm conclusions regarding the benefit of this intervention should be avoided. The only comparison group was a healthy control group; there was no intervention comparison group. This was a feasibility study to determine if an exercise intervention for PFJ OA with a hip and core muscle strengthening and stabilization focus should be explored in future randomized controlled trials. Due to the study design, we cannot make firm conclusions that the exercise intervention was the sole cause of improved symptomatic and functional status of the PFJ OA participants. However, this group of middle-aged women with painful PFJ OA improved their pain intensity, symptoms, ADL, physical performance, QOL, and hip external rotator muscle strength in only 5–6 weeks. The lack of use of a RM testing or a rating of perceived exertion to determine muscle strength baseline and proper exercise intensity, as described by the ACSM, limits our ability to determine if the resistive exercise strengthening program for the hip external rotators, hip extensors, and hip abductors was adequate to result in muscle strengthening [105, 107]. An additional limitation is that the study did not include any men, although it was open to both sexes. Potential participants of both sexes were screened for participation, but the one male screened was excluded due to co-existing neurological and orthopedic conditions. It may be that participants were all female since symptomatic PFJ OA is more prevalent in females than males [11] or because females report greater knee pain, which may make them more interested in treatment [21]. This may limit generalizability of our results to middle-aged females with PFJ OA. The significant difference between the PFJ OA and control groups for BMI is a limitation of this study since elevated BMI is associated with PFJ OA and patellofemoral pain [109]. Future randomized controlled studies examining interventions for PFJ OA should consider matching groups according to BMI to avoid this limitation. Finally, although adherence to supervised exercise sessions over the 6-week intervention was good, the long-term retention rate over 6 months and willingness to respond to a 6-month follow-up questionnaire were fair, while recording home exercise participation in an exercise calendar diary and returning this diary was poor. We are therefore unable to comment on the impact of performance of a home exercise program on participants’ improvement. Future studies should consider additional methods to improve and monitor adherence to the home exercise program, such as checking exercise diaries at each supervised exercise session, use of automated software applications for more frequent exercise reminders and/or for participant recording of exercise sessions, education regarding the benefits of exercise and physical activity for knee OA and patellofemoral pain, and greater variety in exercise programs to improve enjoyment of exercise [110–112].

## Conclusions

This pilot feasibility study demonstrated that a hip and lumbopelvic-hip core muscle strengthening and core stability exercise program is feasible as an intervention for persons with painful PFJ OA. Participants found the exercise program acceptable and were adherent to supervised sessions. Long-term retention at a 6-month follow-up was fair and return of exercise calendar diaries was poor. The intervention appeared to result in improved pain, function, physical performance, hip external rotator muscle strength, and QOL in the short term. Long-term results 6 months after conclusion of the intervention were no longer statistically significant but reported pain, function, and QOL were still above minimum important difference levels. The study shows that an intervention with hip muscle strengthening and core stabilization is appropriate to be studied in future randomized controlled trials.

## Additional file

Additional file 1: Patellofemoral joint osteoarthritis hip and lumbopelvic-hip core muscle exercise program. (DOCX 21 kb)

## Abbreviations

10-RM: 10 repetition maximum
1-RM: 1 repetition maximum
ADL: Activity of daily living
BMI: Body mass index
GRC: Global rating of change
IQR: Interquartile range
IRB: Institutional Review Board
KOOS: Knee Injury and Osteoarthritis Outcome Score
LE: Lower extremity
MCID: Minimum clinically important difference
NPRS: Numeric pain rating scale
OA: Osteoarthritis
OARSI: Osteoarthritis Research Society International
PFJ: Patellofemoral joint
PFP: Patellofemoral pain
QOL: Quality of life
RM: Repetition maximum
SD: Standard deviation
TFJ: Tibiofemoral joint
TUG: Timed-Up-and-Go

## References

[1] Muraki S, Akune T, Oka H, En-yo Y, Yoshida M, Saika A, Suzuki T, Yoshida H, Ishibashi H, Tokimura F, Yamamoto S, Nakamura K, Kawaguchi H, Yoshimura N (2010). Association of radiographic and symptomatic knee osteoarthritis with health-related quality of life in a population-based cohort study in Japan: the ROAD study. Osteoarthr Cartil.

[2] Cross M, Smith E, Hoy D, Nolte S, Ackerman I, Fransen M, Bridgett L, Williams S, Guillemin F, Hill CL, Laslett LL, Jones G, Cicuttini F, Osborne R, Vos T, Buchbinder R, Woolf A, March L (2014). The global burden of hip and knee osteoarthritis: estimates from the global burden of disease 2010 study. Ann Rheum Dis.

[3] Nguyen USDT, Zhang Y, Zhu Y, Niu J, Zhang B, Aliabadi P, Felson DT (2011). Increasing prevalence of knee pain and symptomatic knee osteoarthritis. Ann Intern Med.

[4] Alkan BM, Fidan F, Tosun A, Ardiçoğlu O (2014). Quality of life and self-reported disability in patients with knee osteoarthritis. Mod Rheumatol.

[5] Lawrence RC, Felson DT, Helmick CG, Arnold LM, Choi H, Deyo RA, Gabriel S, Hirsch R, Hochberg MC, Hunder GG, Jordan JM, Katz JN, Kremers HM, Wolfe F, National Arthritis Data Workgroup (2008). Estimates of the prevalence of arthritis and other rheumatic conditions in the United States. Part II. Arthritis Rheum.

[6] McAlindon TE, Snow S, Cooper C, Dieppe PA (1992). Radiographic patterns of osteoarthritis of the knee joint in the community: the importance of the patellofemoral joint. Ann Rheum Dis.

[7] Duncan RC, Hay EM, Saklatvala J, Croft PR (2006). Prevalence of radiographic osteoarthritis—it all depends on your point of view. Rheumatology (Oxford).

[8] Hinman RS, Lentzos J, Vicenzino B, Crossley KM (2014). Is patellofemoral osteoarthritis common in middle-aged people with chronic patellofemoral pain?. Arthritis Care Res (Hoboken)..

[9] Duncan R, Peat G, Thomas E, Wood L, Hay E, Croft P (2008). How do pain and function vary with compartmental distribution and severity of radiographic knee osteoarthritis?. Rheumatology (Oxford).

[10] Stefanik JJ, Niu J, Gross KD, Roemer FW, Guermazi A, Felson DT (2013). Using magnetic resonance imaging to determine the compartmental prevalence of knee joint structural damage. Osteoarthr Cartil.

[11] Kobayashi S, Pappas E, Fransen M, Refshauge K, Simic M (2016). The prevalence of patellofemoral osteoarthritis: a systematic review and meta-analysis. Osteoarthr Cartil.

[12] Duncan R, Peat G, Thomas E, Wood L, Hay E, Croft P (2009). Does isolated patellofemoral osteoarthritis matter?. Osteoarthr Cartil.

[13] Hoglund LT, Lockard MA, Barbe MF, Barr-Gillespie AE, Hillstrom HJ, Reinus WR, Song J (2015). Physical performance measurement in persons with patellofemoral osteoarthritis: a pilot study. J Back Musculoskeletal Rehabil.

[14] Peat G, Duncan RC, Wood LR, Thomas E, Muller S (2012). Clinical features of symptomatic patellofemoral joint osteoarthritis. Arthritis Res Ther.

[15] Hinman RS, Crossley KM (2007). Patellofemoral joint osteoarthritis: an important subgroup of knee osteoarthritis. Rheumatology (Oxford).

[16] Hart HF, Ackland DC, Pandy MG, Crossley KM (2012). Quadriceps volumes are reduced in people with patellofemoral joint osteoarthritis. Osteoarthr Cartil.

[17] Cowan SM, Hart HF, Warden SJ, Crossley KM (2015). Infrapatellar fat pad volume is greater in individuals with patellofemoral joint osteoarthritis and associated with pain. Rheumatol Int.

[18] Crossley KM, Stefanik JJ, Selfe J, Collins NJ, Davis IS, Powers CM, McConnell J, Vicenzino B, Bazett-Jones DM, Esculier JF, Morrissey D, Callaghan MJ (2016). 2016 patellofemoral pain consensus statement from the 4th International Patellofemoral Pain Research Retreat, Manchester. Part 1: terminology, definitions, clinical examination, natural history, patellofemoral osteoarthritis and patient-reported outcome measures. Br J Sports Med.

[19] Crossley KM, Schache AG, Ozturk H, Lentzos J, Munanto M, Pandy MG (2018). Pelvic and hip kinematics during walking in people with patellofemoral joint osteoarthritis compared to healthy age-matched controls. Arthritis Care Res (Hoboken).

[20] Hoglund LT, Hillstrom HJ, Barr-Gillespie AE, Lockard MA, Barbe MF, Song J (2014). Frontal plane knee and hip kinematics during sit-to-stand and proximal lower extremity strength in persons with patellofemoral osteoarthritis: a pilot study. J Appl Biomech.

[21] Glass N, Segal NA, Sluka KA, Torner JC, Nevitt MC, Felson DT, Bradley LA, Neogi T, Lewis CE, Frey-Law LA (2014). Examining sex differences in knee pain: the multicenter osteoarthritis study. Osteoarthr Cartil.

[22] Nelson AE, Allen KD, Golightly YM, Goode AP, Jordan JM (2014). A systematic review of recommendations and guidelines for the management of osteoarthritis: the chronic osteoarthritis management initiative of the U.S. bone and joint initiative. Semin Arthritis Rheum.

[23] Larmer PJ, Reay ND, Aubert ER, Kersten P (2014). Systematic review of guidelines for the physical management of osteoarthritis. Arch Phys Med Rehabil.

[24] McAlindon TE, Bannuru RR, Sullivan MC, Arden NK, Berenbaum F, Bierma-Zeinstra SM, Hawker GA, Henrotin Y, Hunter DJ, Kawaguchi H, Kwoh K, Lohmander S, Rannou F, Roos EM, Underwood M (2014). OARSI guidelines for the non-surgical management of knee osteoarthritis. Osteoarthr Cartil.

[25] Hochberg MC, Altman RD, April KT, Benkhalti M, Guyatt G, McGowan J, Towheed T, Welch V, Wells G, Tugwell P, American College of Rheumatology (2012). American College of Rheumatology 2012 recommendations for the use of nonpharmacologic and pharmacologic therapies in osteoarthritis of the hand, hip, and knee. Arthritis Care Res (Hoboken).

[26] Fernandes L, Hagen KB, Bijlsma JW, Andreassen O, Christensen P, Conaghan PG, Doherty M, Geenen R, Hammond A, Kjeken I, Lohmander LS, Lund H, Mallen CD, Nava T, Oliver S, Pavelka K, Pitsillidou I, da Silva JA, de la Torre J, Zanoli G, Vliet Vlieland TP, European League Against Rheumatism (EULAR) (2013). EULAR recommendations for the non-pharmacological core management of hip and knee osteoarthritis. Ann Rheum Dis.

[27] Waller B, Ogonowska-Slodownik A, Vitor M, Lambeck J, Daly D, Kujala UM, Heinonen A (2014). Effect of therapeutic aquatic exercise on symptoms and function associated with lower limb osteoarthritis: systematic review with meta-analysis. Phys Ther.

[28] Fransen M, McConnell S, Harmer AR, Van der Esch M, Simic M, Bennell KL (2015). Exercise for osteoarthritis of the knee. Cochrane Database Syst Rev.

[29] Juhl C, Christensen R, Roos EM, Zhang W, Lund H (2014). Impact of exercise type and dose on pain and disability in knee osteoarthritis: a systematic review and meta-regression analysis of randomized controlled trials. Arthritis Rheumatol..

[30] Iijima H, Fukutani N, Aoyama T, Fukumoto T, Uritani D, Kaneda E, Ota K, Kuroki H, Matsuda S (2015). Clinical phenotype classifications based on static varus alignment and varus thrust in Japanese patients with medial knee osteoarthritis. Arthritis Rheumatol..

[31] van der Esch M, Knoop J, van der Leeden M, Roorda LD, Lems WF, Knol DL, Dekker J (2015). Clinical phenotypes in patients with knee osteoarthritis: a study in the Amsterdam osteoarthritis cohort. Osteoarthr Cartil.

[32] Kinds MB, Marijnissen AC, Viergever MA, Emans PJ, Lafeber FP, Welsing PM (2013). Identifying phenotypes of knee osteoarthritis by separate quantitative radiographic features may improve patient selection for more targeted treatment. J Rheumatol.

[33] Waarsing JH, Bierma-Zeinstra SM, Weinans H (2015). Distinct subtypes of knee osteoarthritis: data from the osteoarthritis initiative. Rheumatology (Oxford).

[34] Knoop J, van der Leeden M, Thorstensson CA, Roorda LD, Lems WF, Knol DL, Steultjens MP, Dekker J (2011). Identification of phenotypes with different clinical outcomes in knee osteoarthritis: data from the osteoarthritis initiative. Arthritis Care Res (Hoboken)..

[35] Crossley KM, Hinman RS (2011). The patellofemoral joint: the forgotten joint in knee osteoarthritis. Osteoarthr Cartil.

[36] Farrokhi S, Piva SR, Gil AB, Oddis CV, Brooks MM, Fitzgerald GK (2013). Association of severity of coexisting patellofemoral disease with increased impairments and functional limitations in patients with knee osteoarthritis. Arthritis Care Res (Hoboken)..

[37] Mills K, Hunter DJ (2014). Patellofemoral joint osteoarthritis: an individualised pathomechanical approach to management. Best Pract Res Clin Rheumatol.

[38] Bennell KL, Kyriakides M, Metcalf B, Egerton T, Wrigley TV, Hodges PW, Hunt MA, Roos EM, Forbes A, Ageberg E, Hinman RS (2014). Neuromuscular versus quadriceps strengthening exercise in patients with medial knee osteoarthritis and varus malalignment: a randomized controlled trial. Arthritis Rheumatol.

[39] Cho Y, Kim M, Lee W (2015). Effect of proprioceptive training on foot posture, lower limb alignment, and knee adduction moment in patients with degenerative knee osteoarthritis: a randomized controlled trial. J Phys Ther Sci.

[40] Ageberg E, Nilsdotter A, Kosek E, Roos EM (2013). Effects of neuromuscular training (NEMEX-TJR) on patient-reported outcomes and physical function in severe primary hip or knee osteoarthritis: a controlled before-and-after study. BMC Musculoskelet Disord.

[41] Hunt MA, Keefe FJ, Bryant C, Metcalf BR, Ahamed Y, Nicholas MK, Bennell KL (2013). A physiotherapist-delivered, combined exercise and pain coping skills training intervention for individuals with knee osteoarthritis: a pilot study. Knee.

[42] Quilty B, Tucker M, Campbell R, Dieppe P (2003). Physiotherapy, including quadriceps exercises and patellar taping, for knee osteoarthritis with predominant patello-femoral joint involvement: randomized controlled trial. J Rheumatol.

[43] Crossley KM, Marino GP, Macilquham MD, Schache AG, Hinman RS (2009). Can patellar tape reduce the patellar malalignment and pain associated with patellofemoral osteoarthritis?. Arthritis Rheum.

[44] Hunter DJ, Harvey W, Gross KD, Felson D, McCree P, Li L, Hirko K, Zhang B, Bennell K (2011). A randomized trial of patellofemoral bracing for treatment of patellofemoral osteoarthritis. Osteoarthr Cartil.

[45] Crossley KM, Vicenzino B, Lentzos J, Schache AG, Pandy MG, Ozturk H, Hinman RS (2015). Exercise, education, manual-therapy and taping compared to education for patellofemoral osteoarthritis: a blinded, randomised clinical trial. Osteoarthr Cartil.

[46] Callaghan MJ, Parkes MJ, Hutchinson CE, Gait AD, Forsythe LM, Marjanovic EJ, Lunt M, Felson DT (2015). A randomised trial of a brace for patellofemoral osteoarthritis targeting knee pain and bone marrow lesions. Ann Rheum Dis.

[47] Fukuda TY, Melo WP, Zaffalon BM, Rossetto FM, Magalhães E, Bryk FF, Martin RL (2012). Hip posterolateral musculature strengthening in sedentary women with patellofemoral pain syndrome: a randomized controlled clinical trial with 1-year follow-up. J Orthop Sports Phys Ther..

[48] Kooiker L, Van De Port IG, Weir A, Moen MH (2014). Effects of physical therapist-guided quadriceps-strengthening exercises for the treatment of patellofemoral pain syndrome: a systematic review. J Orthop Sports Phys Ther..

[49] Khayambashi K, Mohammadkhani Z, Ghaznavi K, Lyle MA, Powers CM (2012). The effects of isolated hip abductor and external rotator muscle strengthening on pain, health status, and hip strength in females with patellofemoral pain: a randomized controlled trial. J Orthop Sports Phys Ther..

[50] Khayambashi K, Fallah A, Movahedi A, Bagwell J, Powers C (2014). Posterolateral hip muscle strengthening versus quadriceps strengthening for patellofemoral pain: a comparative control trial. Arch Phys Med Rehabil.

[51] Nakagawa TH, Muniz TB, Baldon Rde M, Dias Maciel C, de Menezes Reiff RB, Serrao FV (2008). The effect of additional strengthening of hip abductor and lateral rotator muscles in patellofemoral pain syndrome: a randomized controlled pilot study. Clin Rehabil.

[52] Baldon Rde M, Serrao FV, Scattone Silva R, Piva SR (2014). Effects of functional stabilization training on pain, function, and lower extremity biomechanics in women with patellofemoral pain: a randomized clinical trial. J Orthop Sports Phys Ther..

[53] Alba-Martin P, Gallego-Izquierdo T, Plaza-Manzano G, Romero-Franco N, Nunez-Nagy S, Pecos-Martin D (2015). Effectiveness of therapeutic physical exercise in the treatment of patellofemoral pain syndrome: a systematic review. J Phys Ther Sci.

[54] Peters JS, Tyson NL (2013). Proximal exercises are effective in treating patellofemoral pain syndrome: a systematic review. Int J Sports Phys Ther.

[55] van der Heijden RA, Lankhorst NE, van Linschoten R, Bierma-Zeinstra SM, van Middelkoop M (2015). Exercise for treating patellofemoral pain syndrome. Cochrane Database Syst Rev.

[56] Thomson C, Krouwel O, Kuisma R, Hebron C (2016). The outcome of hip exercise in patellofemoral pain: a systematic review. Man Ther.

[57] Utting MR, Davies G, Newman JH (2005). Is anterior knee pain a predisposing factor to patellofemoral osteoarthritis?. Knee.

[58] Conchie H, Clark D, Metcalfe A, Eldridge J, Whitehouse M (2016). Adolescent knee pain and patellar dislocations are associated with patellofemoral osteoarthritis in adulthood: a case control study. Knee.

[59] Prins MR, van der Wurff P (2009). Females with patellofemoral pain syndrome have weak hip muscles: a systematic review. Aust J Physiother.

[60] Baker KR, Xu L, Zhang Y, Nevitt M, Niu J, Aliabadi P, Yu W, Felson D (2004). Quadriceps weakness and its relationship to tibiofemoral and patellofemoral knee osteoarthritis in Chinese: the Beijing osteoarthritis study. Arthritis Rheum.

[61] Stefanik JJ, Guermazi A, Zhu Y, Zumwalt AC, Gross KD, Clancy M, Lynch JA, Segal NA, Lewis CE, Roemer FW, Powers CM, Felson DT (2011). Quadriceps weakness, patella alta, and structural features of patellofemoral osteoarthritis. Arthritis Care Res (Hoboken)..

[62] Kaya D, Citaker S, Kerimoglu U, Atay OA, Nyland J, Callaghan M, Yakut Y, Yuksel I, Doral MN (2011). Women with patellofemoral pain syndrome have quadriceps femoris volume and strength deficiency. Knee Surg Sports Traumatol Arthrosc.

[63] Wyndow N, Collins N, Vicenzino B, Tucker K, Crossley K (2016). Is there a biomechanical link between patellofemoral pain and osteoarthritis?. A Narrative Review Sports Med.

[64] Biabanimoghadam M, Motealleh A, Cowan SM (2016). Core muscle recruitment pattern during voluntary heel raises is different between patients with patellofemoral pain and healthy individuals. Knee.

[65] Rojhani Shirazi Z, Biabani Moghaddam M, Motealleh A (2014). Comparative evaluation of core muscle recruitment pattern in response to sudden external perturbations in patients with patellofemoral pain syndrome and healthy subjects. Arch Phys Med Rehabil.

[66] Yilmaz Yelvar GD, Cirak Y, Dalkilinc M, Demir YP, Baltaci G, Komurcu M, Yelvar GD. Impairments of postural stability, core endurance, fall index and functional mobility skills in patients with patello femoral pain syndrome. J Back Musculoskelet Rehabil. 2017;30(1):163–70. 10.3233/BMR-160729 27392843

[67] Arazpour M, Bahramian F, Abutorabi A, Nourbakhsh ST, Alidousti A, Aslani H (2016). The effect of patellofemoral pain syndrome on gait parameters: a literature review. Arch Bone Jt Surg.

[68] Lack S, Barton C, Sohan O, Crossley K, Morrissey D (2015). Proximal muscle rehabilitation is effective for patellofemoral pain: a systematic review with meta-analysis. Br J Sports Med.

[69] Ferber R, Bolgla L, Earl-Boehm JE, Emery C, Hamstra-Wright K (2015). Strengthening of the hip and core versus knee muscles for the treatment of patellofemoral pain: a multicenter randomized controlled trial. J Athl Train.

[70] Dolak KL, Silkman C, Medina McKeon J, Hosey RG, Lattermann C, Uhl TL (2011). Hip strengthening prior to functional exercises reduces pain sooner than quadriceps strengthening in females with patellofemoral pain syndrome: a randomized clinical trial. J Orthop Sports Phys Ther..

[71] Boling MC, Bolgla LA, Mattacola CG, Uhl TL, Hosey RG (2006). Outcomes of a weight-bearing rehabilitation program for patients diagnosed with patellofemoral pain syndrome. Arch Phys Med Rehabil.

[72] Earl JE, Hoch AZ (2011). A proximal strengthening program improves pain, function, and biomechanics in women with patellofemoral pain syndrome. Am J Sports Med.

[73] Lowry CD, Cleland JA, Dyke K (2008). Management of patients with patellofemoral pain syndrome using a multimodal approach: a case series. J Orthop Sports Phys Ther.

[74] Tyler TF, Nicholas SJ, Mullaney MJ, MP MH (2006). The role of hip muscle function in the treatment of patellofemoral pain syndrome. Am J Sports Med.

[75] Yilmaz Yelvar GD, Baltaci G, Bayrakci Tunay V, Atay AO (2015). The effect of postural stabilization exercises on pain and function in females with patellofemoral pain syndrome. Acta Orthop Traumatol Turc.

[76] Willson JD, Dougherty CP, Ireland ML, Davis IM (2005). Core stability and its relationship to lower extremity function and injury. J Am Acad Orthop Surg.

[77] Powers CM, Witvrouw E, Davis IS, Crossley KM (2017). Evidence-based framework for a pathomechanical model of patellofemoral pain: 2017 patellofemoral pain consensus statement from the 4th International Patellofemoral Pain Research Retreat, Manchester, UK: part 3. Br J Sports Med.

[78] Brantingham JW, Globe GA, Cassa TK, Globe D, de Luca K, Pollard H, Lee F, Bates C, Jensen M, Mayer S, Korporaal C (2010). A single-group pretest posttest design using full kinetic chain manipulative therapy with rehabilitation in the treatment of 18 patients with hip osteoarthritis. J Manip Physiol Ther.

[79] Altman RD, Hochberg M, Murphy WA, Wolfe F, Lequesne M (1995). Atlas of individual radiographic features in osteoarthritis. Osteoarthr Cartil.

[80] Hart DJ, Spector TD (1995). The classification and assessment of osteoarthritis. Baillieres Clin Rheumatol.

[81] Sanders TL, Pareek A, Hewett TE, Stuart MJ, Dahm DL, Krych AJ. High rate of recurrent patellar dislocation in skeletally immature patients: a long-term population-based study. Knee Surg Sports Traumatol Arthrosc. 2017; 10.1007/s00167-017-4505-y. 10.1007/s00167-017-4505-y 28299386

[82] Halket A, Stratford PW, Kennedy DM, Woodhouse LJ, Spadoni G (2008). Measurement properties of performance-specific pain ratings of patients awaiting total joint arthroplasty as a consequence of osteoarthritis. Physiother Can.

[83] Ruyssen-Witrand A, Fernandez-Lopez CJ, Gossec L, Anract P, Courpied JP, Dougados M (2011). Psychometric properties of the OARSI/OMERACT osteoarthritis pain and functional impairment scales: ICOAP, KOOS-PS and HOOS-PS. Clin Exp Rheumatol.

[84] Farrar JT, Young JP, LaMoreaux L, Werth JL, Poole RM (2001). Clinical importance of changes in chronic pain intensity measured on an 11-point numerical pain rating scale. Pain.

[85] Roos EM, Roos HP, Lohmander LS, Ekdahl C, Beynnon BD (1998). Knee injury and osteoarthritis outcome score (KOOS)—development of a self-administered outcome measure. J Orthop Sports Phys Ther..

[86] Collins NJ, Prinsen CA, Christensen R, Bartels EM, Terwee CB, Roos EM (2016). Knee Injury and Osteoarthritis Outcome Score (KOOS): systematic review and meta-analysis of measurement properties. Osteoarthr Cartil.

[87] Roos EM, Lohmander LS (2003). The Knee injury and Osteoarthritis Outcome Score (KOOS): from joint injury to osteoarthritis. Health Qual Life Outcomes.

[88] Podsiadlo D, Richardson S (1991). The timed “Up & Go”: a test of basic functional mobility for frail elderly persons. J Am Geriatr Soc.

[89] French HP, Fitzpatrick M, FitzGerald O (2011). Responsiveness of physical function outcomes following physiotherapy intervention for osteoarthritis of the knee: an outcome comparison study. Physiotherapy.

[90] Dobson F, Hinman RS, Hall M, Terwee CB, Roos EM, Bennell KL (2012). Measurement properties of performance-based measures to assess physical function in hip and knee osteoarthritis: a systematic review. Osteoarthr Cartil.

[91] Dobson F, Hinman RS, Roos EM, Abbott JH, Stratford P, Davis AM, Buchbinder R, Snyder-Mackler L, Henrotin Y, Thumboo J, Hansen P, Bennell KL (2013). OARSI recommended performance-based tests to assess physical function in people diagnosed with hip or knee osteoarthritis. Osteoarthr Cartil.

[92] Stratford PW, Kennedy DM, Woodhouse LJ (2006). Performance measures provide assessments of pain and function in people with advanced osteoarthritis of the hip or knee. Phys Ther.

[93] Smith JA, Popovich JM, Kulig K (2014). The influence of hip strength on lower-limb, pelvis, and trunk kinematics and coordination patterns during walking and hopping in healthy women. J Orthop Sports Phys Ther..

[94] Souza RB, Powers CM (2009). Differences in hip kinematics, muscle strength, and muscle activation between subjects with and without patellofemoral pain. J Orthop Sports Phys Ther..

[95] Popovich JM, Kulig K (2012). Lumbopelvic landing kinematics and EMG in women with contrasting hip strength. Med Sci Sports Exerc.

[96] Hoglund LT, Wong AL, Rickards C (2014). The impact of sagittal plane hip position on isometric force of hip external rotator and internal rotator muscles in healthy young adults. Int J Sports Phys Ther..

[97] Bazett-Jones DM, Cobb SC, Joshi MN, Cashin SE, Earl JE (2011). Normalizing hip muscle strength: establishing body-size-independent measurements. Arch Phys Med Rehabil.

[98] Stauber WT, Barill ER, Stauber RE, Miller GR (2000). Isotonic dynamometry for the assessment of power and fatigue in the knee extensor muscles of females. Clin Physiol.

[99] Lankhorst NE, Bierma-Zeinstra SM, van Middelkoop M (2013). Factors associated with patellofemoral pain syndrome: a systematic review. Br J Sports Med.

[100] Crossley KM, Vicenzino B, Pandy MG, Schache AG, Hinman RS (2008). Targeted physiotherapy for patellofemoral joint osteoarthritis: a protocol for a randomised, single-blind controlled trial. BMC Musculoskelet Disord.

[101] Kamper SJ, Maher CG, Mackay G (2009). Global rating of change scales: a review of strengths and weaknesses and considerations for design. J Man Manip Ther.

[102] Kemp JL, Schache AG, Makdissi M, Sims KJ, Crossley KM (2013). Greater understanding of normal hip physical function may guide clinicians in providing targeted rehabilitation programmes. J Sci Med Sport.

[103] Bartholdy C, Juhl C, Christensen R, Lund H, Zhang W, Henriksen M (2017). The role of muscle strengthening in exercise therapy for knee osteoarthritis: a systematic review and meta-regression analysis of randomized trials. Semin Arthritis Rheum.

[104] Dobson F, Bennell KL, French SD, Nicolson PJ, Klaasman RN, Holden MA, Atkins L, Hinman RS (2016). Barriers and facilitators to exercise participation in people with hip and/or knee osteoarthritis: synthesis of the literature using behavior change theory. Am J Phys Med Rehabil.

[105] Minshull C, Gleeson N (2017). Considerations of the principles of resistance training in exercise studies for the management of knee osteoarthritis: a systematic review. Arch Phys Med Rehabil.

[106] Ellis MI, Seedhom BB, Wright V (1984). Forces in the knee joint whilst rising from a seated position. J Biomed Eng.

[107] Riebe D, Pescatello LS (2014). General principles of exercise prescription. ACSM’s guidelines for exercise testing and prescription.

[108] Peterson MD, Gordon PM (2011). Resistance exercise for the aging adult: clinical implications and prescription guidelines. Am J Med.

[109] Hart HF, Barton CJ, Khan KM, Riel H, Crossley KM (2017). Is body mass index associated with patellofemoral pain and patellofemoral osteoarthritis? A systematic review and meta-regression and analysis. Br J Sports Med.

[110] Rathleff MS, Roos EM, Olesen JL, Rasmussen S (2015). Exercise during school hours when added to patient education improves outcome for 2 years in adolescent patellofemoral pain: a cluster randomised trial. Br J Sports Med.

[111] Barton CJ, Lack S, Hemmings S, Tufail S, Morrissey D (2015). The ‘best practice guide to conservative management of patellofemoral pain’: incorporating level 1 evidence with expert clinical reasoning. Br J Sports Med.

[112] Dishman RK, Jackson AS, Bray MS (2014). Self-regulation of exercise behavior in the TIGER study. Ann Behav Med.

