# Supplementary material for: A 6-week hip muscle strengthening and lumbopelvic-hip core stabilization program to improve pain, function, and quality of life in persons with patellofemoral osteoarthritis: a feasibility pilot study
Source: Pilot Feasibility Stud. 2018 Apr 6;4:70. doi: 10.1186/s40814-018-0262-z (PMC5889597; doi:10.1186/s40814-018-0262-z)
Supplement: Supplementary file 1 — Patellofemoral joint osteoarthritis hip and lumbopelvic-hip core muscle exercise program. (DOCX 21 kb) [file 40814_2018_262_MOESM1_ESM.docx]

Patellofemoral Joint Osteoarthritis Hip and Lumbopelvic-Hip Core Muscle Exercise Program

*Exercise Program: In-Clinic*

Stretches at End of Session (all sessions): seated hamstrings, prone quadriceps, standing gastrocnemius stretches (2 repetitions/30 second hold per muscle group bilaterally)

Week 1 (1 session)

1. Prone transversus abdominis (TA) isometric (Stabilizer for biofeedback): 10 repetitions/5 second hold
2. Prone gluteus maximus isometric with knee flexed: 10 repetitions/5 second hold
3. Hooklying TA isometrics with alternate hip/knee flexion/extension (Stabilizer for biofeedback): 3 sets of 10 repetitions
4. Sidelying hip external rotation (ER) with feet together (clamshells): 3 sets of 10 repetitions
5. Sidelying hip abduction (ABD): 3 sets of 10 repetitions
6. Single-leg stance (SLS) with maintenance of level pelvis (visual and verbal feedback): 10 repetitions/5 second hold

Week 2 (2 sessions)

1. Hooklying TA isometrics with alternate hip/knee flexion/extension (Stabilizer for biofeedback): 3 sets of 10 repetitions
2. Bridging with TA isometric: 3 sets of 10 repetitions
3. Clamshells with focus on eccentric phase: 3 sets of 10 repetitions/5 second lowering
4. Sidelying hip ABD: focus on eccentric phase: 3 sets of 10 repetitions/5 second lowering
5. Prone hip extension (EXT) with knee flexed to 90 degrees (on 1 pillow, maintain TA contraction to avoid lumbar EXT): 3 sets of 10 repetitions
6. Seated hip ER with theraband resistance at ankle: 3 sets of 10 repetitions
7. SLS with level pelvis adding contralateral upper extremity raises in sagittal plane (visual and verbal feedback): 2 sets of 10 repetitions

Week 3 (2 sessions)

1. Bridging with TA isometric and hip ABD/ER (theraband around distal thighs): 3 sets of 10 repetitions
2. Prone on elbows and knees “planks”: isometric core stabilization: 10 repetitions/10 second hold
3. Clamshells with added hip ABD (lift upper foot off lower foot, maintain hip ER), eccentric focus: 3 sets of 10 repetitions
4. Sidelying hip ABD with cuff weight resistance, eccentric focus: 3 sets of 10 repetitions
5. Prone hip EXT with knee flexed to 90 degrees (on 1 pillow, TA contraction), eccentric focus with cuff resistance: 3 sets of 10 repetitions
6. SLS with level pelvis with contralateral theraband pulls (visual feedback only): resisted shoulder flexion and extension: 2 sets of 10 repetitions

Week 4 (2 sessions)

1. Clamshells with hip ABD with cuff weight resistance at distal thigh, eccentric focus: 3 sets of 10 repetitions
2. Prone elbow/knee planks: 10 repetitions/10 second hold
3. Side planks on one elbow and knees (knees at 90 degrees flexion): 5 repetitions per side/10 second hold
4. Bridging 2 🡪1 foot support (extend knee) with level pelvis and knee of supporting limb over foot: 1 set of 10 repetitions/side
5. SLS with level pelvis with contralateral hip ABD (visual feedback only): 2 sets of 10 repetitions/side
6. Control of hip/knee during forward step (maintain knee over center of foot – visual and verbal feedback): 2 sets of 10 repetitions/side

Week 5 (2 sessions)

1. Prone elbow/knee planks: 10 repetitions/20 second hold
2. Side planks on one elbow and knees (knees at 90 degrees flexion): 5 repetitions per side/10 second hold
3. SLS with level pelvis with contralateral hip EXT: 2 sets of 10 repetitions/side
4. SLS on step (contralateral foot off step in lateral stepping position): controlled pelvic lowering/raising (eccentric focus on hip ABD): 1 set of 10 repetitions/side
5. Stand 🡪 sit on elevated seat with knees over center of feet (0 🡪 45 degrees knee flexion): 1 set of 10 repetitions
6. Forward partial lunge (maintain knee over center of foot): 2 sets of 10 repetitions/side

Week 6 (1 session + home exercise program review/maintenance exercise program instruction at 2^nd^ session)

1. Prone planks (on elbows and toes): 10 repetitions/10 second hold
2. Side planks on one elbow and feet: 5 repetitions per side/5 second hold
3. SLS with level pelvis with contralateral hip EXT against theraband: 2 sets of 10 repetitions/side
4. SLS with level pelvis with contralateral hip ABD against theraband: 2 sets of 10 repetitions/side
5. Stand 🡪 sit on elevated seat with knees over center of feet (0 🡪 45 degrees knee flexion) with theraband around distal thighs (resist hip ER/ABD): 1 set of 10 repetitions
6. Forward partial lunge (maintain knee over center of foot): 2 sets of 10 repetitions/side

*Exercise Program Resistance and Repetitions*

Week 1 exercises were performed without resistance. The focus of Week 1 was on proper performance of exercises with good lumbopelvic-hip core stabilization. Resistance level and repetitions/sets were customized for each participant according to 1) ability to perform the exercises with good core stabilization as determined by a) Spinal Stabilizer biofeedback unit readings and b) visual observation by the examiner; 2) reported fatigue and/or soreness level of the participant; 3) the presence of comorbidities (e.g., history of shoulder instability and pain limiting ability to perform plank exercises). Resistance was added beginning in Week 2 with resistive bands (Theraband™) and with cuff weights, as appropriate. When resistance was increased, the number of repetitions in a set was decreased to 6-8 repetitions, according to each participant reported ability to perform the set. The goal was to increase to 10 repetitions per set by the second supervised exercise session of each week.

Participants were not forced to advance exercises if they were unable to perform the new exercises with good control and without knee pain increase (or pain production in other regions, for example, shoulder pain in side planks).

*Exercise Program: At Home*

Participants were provided with pictorial and written descriptions of exercises identical or similar to those performed in the clinic. The home exercise program mirrored the in clinic program. Participants were instructed to perform the home program once daily on days not travelling to the clinic for the supervised session. They were given Theraband™ of an appropriate resistive level to use in their exercises at home; those exercises using cuff weights were performed without resistance. In addition, beginning with week 5 they were instructed to walk for 20-30 minutes twice weekly. Participants already walking for fitness were not forced to stop this activity or to reduce frequency of walking.

*Maintenance Home Exercise Program*

Participants were instructed to continue the exercise program for Week 6 on a thrice weekly basis for the maintenance program. Participants were encouraged to purchase cuff weights to use in maintenance home exercise programs and provided with appropriate resistive amounts for each exercise. They were also instructed to walk for 20-30 minutes twice weekly and instructed in target heart rate to achieve 60-85% of maximal permissible heart rate (220-age).
